# Modeling of Heat Stress in Sows—Part 1: Establishment of the Prediction Model for the Equivalent Temperature Index of the Sows

**DOI:** 10.3390/ani11051472

**Published:** 2021-05-20

**Authors:** Mengbing Cao, Chao Zong, Xiaoshuai Wang, Guanghui Teng, Yanrong Zhuang, Kaidong Lei

**Affiliations:** 1 College of Water Resources and Civil Engineering, China Agricultural University, Beijing 100083, China; mengbing-cao@cau.edu.cn (M.C.); futong@cau.edu.cn (G.T.); zyr123@cau.edu.cn (Y.Z.); leikaidong@cau.edu.cn (K.L.); 2 Key Laboratory of Agricultural Engineering in Structure and Environment, Ministry of Agriculture and Rural Affairs, Beijing 100083, China; 3 College of Biosystems Engineering and Food Science, Zhejiang University, No. 866 Yuhangtang Road, Hangzhou 310058, China; xiaoshuai.wang@hotmail.com

**Keywords:** thermal index, sows, environmental parameters, heat stress threshold, skin temperature

## Abstract

**Simple Summary:**

Sows are susceptible to heat stress. Various indicators can be found in the literature assessing the level of heat stress in pigs, but none of them is specific to assess the sows’ thermal condition. Moreover, previous thermal indices have been developed by considering only partial environment parameters, and the interaction between the index and the animal’s physiological response are not always included. Therefore, this study aims to develop and assess a new thermal index specified for sows, called equivalent temperature index for sows (ETIS), with a comprehensive consideration of the influencing factors. An experiment was conducted, and the experimental data was applied for model development and validation. The equivalent temperatures have been transformed on the basis of equal effects of air velocity, relative humidity, floor heat conduction and indoor radiation on the thermal index, and used for the ETIS combination. The correlations between ETIS and sow’s physiological parameters were performed. In the comparison with other thermal indices, the ETIS had the best performance (R = 0.82) using experimental data obtained from the sow house. In addition, the comfort threshold of ETIS has been classified for evaluating heat stress levels in the sow. This study concludes that the newly developed ETIS can be used to assess the degree of thermal comfort for sows.

**Abstract:**

Heat stress affects the estrus time and conception rate of sows. Compared with other life stages of pigs, sows are more susceptible to heat stress because of their increased heat production. Various indicators can be found in the literature assessing the level of heat stress in pigs. However, none of them is specific to assess the sows’ thermal condition. Moreover, thermal indices are mainly developed by considering partial environment parameters, and there is no interaction between the index and the animal’s physiological response. Therefore, this study aims to develop a thermal index specified for sows, called equivalent temperature index for sows (ETIS), which includes parameters of air temperature, relative humidity and air velocity. Based on the heat transfer characteristics of sows, multiple regression analysis is used to combine air temperature, relative humidity and air velocity. Environmental data are used as independent variables, and physiological parameters are used as dependent variables. In 1029 sets of data, 70% of the data is used as the training set, and 30% of the data is used as the test set to create and develop a new thermal index. According to the correlation equation between ETIS and temperature-humidity index (THI), combined with the threshold of THI, ETIS was divided into thresholds. The results show that the ETIS heat stress threshold is classified as follows: suitable temperature ETIS < 33.1 °C, mild temperature 33.1 °C ≤ ETIS < 34.5 °C, moderate stress temperature 34.5 °C ≤ ETIS < 35.9 °C, and severe temperature ETIS ≥ 35.9 °C. The ETIS model can predict the sows’ physiological response in a good manner. The correlation coefficients R of skin temperature was 0.82. Compared to early developed thermal indices, ETIS has the best predictive effect on skin temperature. This index could be a useful tool for assessing the thermal environment to ensure thermal comfort for sows.

## 1. Introduction

Heat stress can affect the reproductive endocrine system in sows and inhibit the ovarian function, which in turn affects estrus activity [1], causing delayed estrus, hidden estrus or even no estrus phenomenon [2]. Heat stress can reduce fertility rate [3,4], decrease piglet weight gain [5], decrease milk production [6], increase weight loss through lactation [5] and increase death rate [7]. According to reports, the annual economic losses caused by heat stress to the pig industry amounted to 299 million dollars in the US, and the number reached billions of dollars globally [8]. Sows’ performance can greatly affect the profitable margin of a farm, and proper means to reduce heat stress are desperately needed. In order to alleviate heat stress of sows more effectively, it is necessary to quantify the thermal environment of the sow barn.

Over the past decades, many indices have been developed in the assessment of thermal environment, and some have been applied with pigs, such as the temperature-humidity index (THI) [9,10,11,12,13,14,15,16], the globe-humidity index (BGHI) [17,18], the effective temperature (ET) [19,20] and the enthalpy (H) [21]. However, those indices applied with pigs contain the following issues: (1) they normally include two or three environmental parameters, which are unilateral from the perspective of heat exchange [22,23]; (2) those indices mainly developed based on other animals, while directly applied in pigs; (3) they lack consideration of pigs’ real-time physiological and production characteristics. In addition, there is no study focusing on heat stress in sows, so far. To overcome the limitation of those indices, a specific thermal index developed for sows is necessary.

Respiratory rate, core body temperature, rectal temperature, skin temperature, feed, water intake and other physiological responses as well as production performance (pregnancy rate, delivery times, estrus time, etc.) are affected by heat stress to varying levels [24,25,26,27,28]. Thus, the level of heat stress can be assessed by measuring or monitoring changes in physiological responses, animal behavior and performance. However, animal behavior, performance and most physiological indicators are either invasive or difficult to monitor continuously. In contrast, environmental parameters, such as air temperature, relative humidity and air velocity, acting as the influencing factors of heat stress, are comparatively much easier to measure. Therefore, it is necessary to research the relationship between environmental parameters and animal physiological responses, and establish a heat stress index.

The thermal index, which includes environmental parameters such as air temperature, relative humidity and air velocity, is often used to analyze heat stress in animals. The total heat dissipation of the sow will be affected by the sow’s convective heat transfer, radiation heat transfer, heat conduction and respiratory heat transfer [22,23,29], and those forms of heat dissipation will be affected by environmental factors such as air temperature, relative humidity airflow velocity and so on. Heat stress of sows is mainly caused by poor heat dissipation [22,30,31]. Therefore, heat dissipation should be considered in the establishment of a thermal index.

Therefore, the objectives of this study are (1) to develop a new thermal index for sows based on environmental parameters and physiological responses; (2) to categorize the developed thermal index with heat stress levels in sows.

## 2. Materials and Methods

### 2.1. Model Development

#### 2.1.1. Structure of the Equivalent Temperature Index for Sows (ETIS) Model

In this study, the new thermal index is expressed as the equivalent temperature index of a sow (ETIS). ETIS is composed of air temperature and equivalent temperatures adjusted from the thermal effects of air velocity, relative humidity, floor heat conduction and radiation on the heat load. Mathematically, ETIS is expressed as

(1) $$\mathrm{ETIS}=T+T_{\mathrm{rh}}+T_{f}+T_{u}+T_{r}$$

where T is the dry-bulb temperature of the air (°C); T_rh_, T_u_, T_f_ and T_r_ are equivalent air temperatures related to relative humidity, air velocity, floor heat conduction and radiation, respectively (°C).

#### 2.1.2. Equivalent Temperature Based on Relative Humidity 

Beckett [32] provides a chart to illustrate the combined effects of air temperature and relative humidity on pig growth. Bjerg [19] compared the values from Beckett’s study and found that T_rh_ can be calculated by Equation (2):

(2) $$T_{\mathrm{rh}}=a\cdot \left(\mathrm{RH}-50\right)\cdot T$$

where T_rh_, is equivalent air temperature related to relative humidity (°C). a is a coefficient, and expected to be positive. RH is the relative humidity (%). T is the dry-bulb temperature of the air (°C) 

It can be concluded from the equation that when the humidity is above 50%, as the humidity increases, the thermal index shows an upward trend.

#### 2.1.3. Equivalent Temperature Based on Air Velocity

The equivalent air velocity temperature can be obtained from the equations related to convective heat transfer [22,33,34], as shown in Equations (3) and (4)

(3) $$H_{c}=h_{c}\cdot A\cdot \left(T_{s}-T\right)$$

(4) $$\mathrm{Nu}=\frac{h_{c}\cdot l}{k}={\mathrm{mRe}}^{c}=m{\left(\frac{\rho \mathrm{ul}}{\mu }\right)}^{c}$$

where H_c_ is total heat transfer rate (W), h_c_ is convective heat transfer coefficient (W·m^−2^·°C^−1^), A is surface area of the animal (m^2^) and T_s_ is skin temperature (°C). Nu is Nusselt number, l is characteristic length (m) and k is air thermal conductivity (W·m^−1^·°C ^−1^). Re is Reynolds number. ρ is air density (kg·m^−3^). u is air velocity (m·s^−1^). μ is dynamic viscosity coefficient (m^2^·s^−1^). m and c are constants determined by the relationship between Nu and Re. 

According to Equations (3) and (4), it can be seen that the air velocity has an extremely important influence on the convective heat transfer coefficient, so the equivalent temperature of air velocity (T_u_) is expressed as Equation (5):

(5) $$T_{u}=e\cdot u^{c}\cdot \left(T_{s}-T\right)$$

where T_u_ is equivalent air temperature related to air velocity (°C). e is a coefficient that represents the relationship between convective heat transfer of the sow and equivalent temperature based on air velocity. u is air velocity (m·s^−1^). c is a constant determined by the relationship between Nu and Re. T_s_ is skin temperature (°C). T is the dry-bulb temperature of the air (°C) 

Equation (5) is consistent with Wang’s equation [35], where the constant c represents the effect of air velocity changes on the convective heat transfer coefficient. This constant is usually obtained by analyzing the relationship between air velocity and object convection heat transfer coefficient [36,37].

Li [36] used computational fluid dynamic (CFD) simulation to study the convective heat transfer of a standing pig and found that it is proportional to v^0.66^. However, the sow spends most of the time lying down and resting [38,39]. To know the convective heat transfer of sows, a pilot study by Cao et al. [40] was carried out, and the convective heat transfer coefficient of sows was found to be 0.6827.

#### 2.1.4. Equivalent Temperature Based on Conductive Heat Transfer

The heat transfer between the sow body and the floor surface is driven by the temperature difference. In this study, a concrete floor instead of slatted floor was installed in the sow barn. In the summer, the surface of the floor is a source of cold. The sow heat can be transferred to the floor surface [20,22]. Then, the equivalent temperature of heat conduction at that place must be related to the temperature of the floor surface and sow body surface, and the relationship between them is indicated by D. Thus, T_f_ can be expressed as Equation (6):

(6) $$T_{f}=D\cdot \left(T_{s}-T_{d}\right)$$

where T_f_ is equivalent air temperature related to floor heat conduction (°C). D is a coefficient related to floor heat conduction equivalent temperature. T_s_ is skin temperature (°C). T_d_ is the surface temperature for the floor (°C). 

As the floor surface temperature is very close to the ambient air temperature, the air temperature is usually used as the floor surface temperature for calculations [41,42]. The equivalent temperature of floor heat conduction can be expressed as

(7) $$T_{f}=D\cdot \left(T_{s}-T\right)$$

where T_f_ is equivalent air temperature related to floor heat conduction (°C). D is a coefficient related to floor heat conduction equivalent temperature. T_s_ is skin temperature (°C). T is the dry-bulb temperature of the air (°C).

#### 2.1.5. Equivalent Temperature Based on Radiative Heat Transfer

The radiative heat transfer is driven by the difference of the fourth power of the absolute temperatures between two objects. For sows in the house, long-wave radiation was considered. To integrate the effects of the radiation between the measured object and the surrounding surfaces, the mean radiant temperature was introduced [23,43,44]. The radiative heat of a sow is related to the sow’s skin temperature and mean radiant temperature, and Equation (8) is obtained:

(8) $$T_{r}=R_{\mathrm{rad}}\cdot \left({\left(T_{s}+273.15\right)}^{4}-{\left(T_{\mathrm{rad}}+273.15\right)}^{4}\right)$$

where T_r_ is equivalent air temperature related to radiation. R_rad_ is a coefficient, which represents the relationship between the sow’s long-wave radiation and the equivalent radiation temperature. T_s_ is skin temperature (°C). T_rad_ is the average radiant temperature (°C).

Since indoor climate studies usually assume that the average radiant temperature is equal to the air temperature [44,45,46,47,48], T_rad_ is represented here by T, and equivalent temperature of radiative heat transfer can be expressed as Equation (9).

(9) $$T_{r}=R_{\mathrm{rad}}\cdot \left({\left(T_{s}+273.15\right)}^{4}-{\left(T+273.15\right)}^{4}\right)$$

where T_r_ is equivalent air temperature related to radiation. R_rad_ is a coefficient, which represents the relationship between the sow’s long-wave radiation and the equivalent radiation temperature. T_s_ is skin temperature (°C). T is the dry-bulb temperature of the air (°C).

#### 2.1.6. Combined Equivalent Temperature Index

The ETIS is formed by integrating those equivalent temperatures into Equation (1) and shown as

ETIS = T + a·(RH − 50)·T + e·u^0.6827^·(T_s_ − T) + D·(T_s_ − T) + R_rad_·((T_s_ + 273.15)^4^ − (T + 273.15)^4^) (10)

where ETIS is the equivalent temperature index for sows. T is the dry-bulb temperature of the air (°C). a is the coefficient related to the relative humidity equivalent temperature. RH is the relative humidity (%). e is a coefficient that represents the relationship between convective heat transfer of the sow and equivalent temperature based on air velocity. u is air velocity (m·s^−1^). T_s_ is skin temperature (°C). D is a coefficient related to floor heat conduction equivalent temperature. R_rad_ is a coefficient, which represents the relationship between the sow’s long-wave radiation and the equivalent radiation temperature.

### 2.2. Experimental Set Up

#### 2.2.1. Animal and Housing

The study was conducted in a sow barn at the National Feed Research Center of China Agricultural University from June to August 2018. Sows were crossbreeds between Large White and Landrace. 

**Figure 1:**
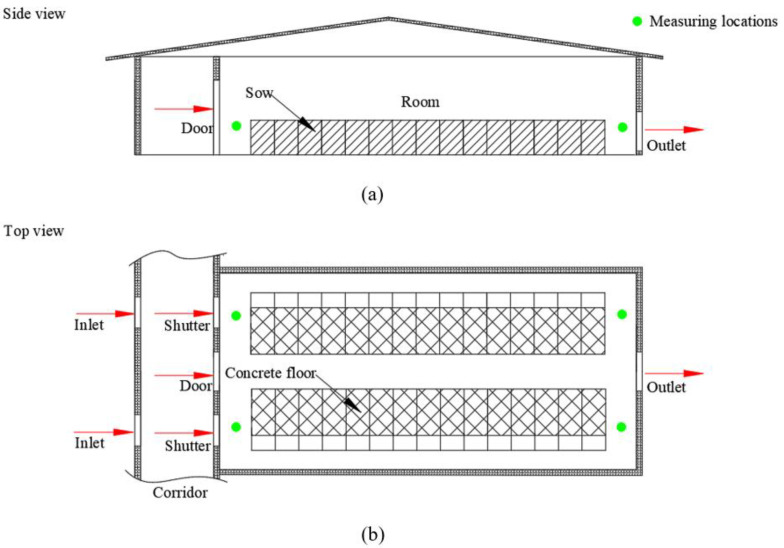
Barn schematic: (**a**) Side view; (**b**) Top view. Barn can house 30 sows, usually 20–24 non-pregnant sows per batch. Sow barn is 11.7 m × 7.7 m × 2.5 m. The length, width and height of the crate is 2.2 m × 0.64 m × 1 m. The sow barn has four measuring points, and each measuring point measures the air temperature, relative humidity and air velocity. This research assumed that the difference between different measuring points in environmental parameters is linear (for example, the green dots in figure (**a**) were set to be 30 °C and 31.5 °C, the environmental parameters for the 15 sows were 30.1, 30.2, 30.3, 30.4, …, 31.5 °C), and the environmental parameters were determined at different sow locations based on this assumption. Physiological parameters of the sows in the air outlet area, the air inlet area, and the middle area of the room were measured.

The barn housed 30 non-pregnant multiparous sows. Sows were fed in this facility before being moved for breeding. The rectal temperature, respiration rate and skin temperature of the sows were measured. Data collection was done during non-pregnancy. Data were collected on each batch of 10 sows and on a total of 4 batches. The sows were kept in crates with concrete solid floors, as shown in Figure 1a,b. The length, width and height of the crate was 2.2 m × 0.64 m × 1 m. Each crate was equipped with one feeder and one drinker, and the sow was raised with ad libitum feeding and drinking. The slurry was removed regularly by workers, and the urine ran into the drain pipe beneath the floor. A tunnel ventilation system with one exhaust fan and 2 air inlets was used in the house. The fan (YH900, Yinghe Company, Shenzhen, China) had a capacity of 28,500 m^3^·h^−1^, and the ventilation rate was controlled based on indoor air temperature.

#### 2.2.2. Measurements

##### Environment

Air velocity, air temperature and relative humidity were measured by wireless hot wire anemometers (testo405i, Schwarzwald, Germany) and a wireless air temperature and relative humidity measuring instrument (testo605i, Schwarzwald, Germany) at 4 locations (Figure 1b). The measuring points were 0.6 m above the floor. During the experiment, a set of data was collected every 2 h from 8:00 to 18:00 per day. The air temperature, relative humidity and air velocity at the measuring point in Figure 1 were measured. The research assumed that the difference between different measuring points in environmental parameters is linear, and the environmental parameters were determined at different locations based on this assumption. Finally, the environmental conditions of sows were determined.

##### Physiological Parameters

The rectal temperature, respiratory rate and skin temperature of the sows in the air outlet area, the air inlet area and the middle area of the room were measured. Physiological parameters were measured on each batch of 10 sows in each of 4 batches during the non-pregnant period. The rectal temperature probe (Huaxu, Xinxiang, China) was used to measure the rectal temperatures of sows. The rate of respiration was calculated by counting the number of rises and falls of the sow’s chest within one minute. The skin temperature was measured by a handheld infrared thermometer (Raynger ST, Raytek, Santa Cruz, California, CA, USA) with an accuracy of ±1% of the measured temperature. The physiological parameters were measured every 2 h from 8:00 to 18:00 every day. The testing of physiological data was performed in parallel with the testing of environmental data. 

### 2.3. Data Analysis

#### 2.3.1. Correlation Analysis between Temperature and Humidity Index and Physiological Parameters

Correlation analysis was used to determine the correlations between THI [10,49] and core temperature, between THI [10,49] and respiration rate, and the correlations between THI [10,49] and skin temperature. According to the correlation analysis between THI and three physiological parameters, the physiological parameter that had a strong connection with the thermal index was determined, and this physiological parameter was used as the dependent variable for creating the ETIS.

#### 2.3.2. Linear Regression Model

In the regression model, instead of using three environmental parameters (T, RH, and u), four new corresponding terms—T, RH, u^0.6827^·(T_S_ − T) and (T + 273.15)^4^—were used as explanatory variables in the model fitting and analysis.

Skin temperature was selected as the only response variable because skin temperature is influenced by both the thermal environment and the sow body, acting as the window for transfer heat.

The linear regression model associated with Equation (10) is now expressed as follows:

y = b_0_ + b_1_·T + b_2_·(50)·T + b_3_·(RH)·T + b_4_·u^0.6827^·(T_s_ − T) + b_5_·T_s_ + b_6_·T + b_7_·(T_s_ + 273.15)^4^ + b_8_·(T + 273.15)^4^ (11)

where y is the response variable (skin temperature); b_0_ is the intercept; b_1_, b_2_, b_3_, b_4_, b_5_, b_6_, b_7_ and b_8_ are the regression coefficients. T is the dry-bulb temperature of the air (°C). RH is the relative humidity (%). u is air velocity (m·s^−1^). T_s_ is skin temperature (°C). 

Since the skin temperature is used as the response variable, T_s_ in Equation (11) is fixed at 38 °C to ensure that physiological parameters are not included in the dependent variable. Equation (11) eventually becomes

(12) $$\begin{array}{l} y=b_{0}+b_{1}\cdot T+b_{2}\cdot \left(50\right)\cdot T+b_{3}\cdot \left(\mathrm{RH}\right)\cdot T+b_{4}\cdot u^{0.6827}\cdot \left(38-T\right)+b_{5}\cdot 38+b_{6}\cdot T+b_{7}\cdot {\left(38+273.15\right)}^{4}\\ \;\;\;\;\;\;+b_{8}\cdot {\left(T+273.15\right)}^{4} \end{array}$$

where y is the response variable (skin temperature); b_0_ is the intercept; b_1_, b_2_, b_3_, b_4_, b_5_, b_6_, b_7_ and b_8_ are the regression coefficients. T is the dry-bulb temperature of the air (°C). RH is the relative humidity (%). u is air velocity (m·s^−1^). 

#### 2.3.3. Regression Analysis

The experimental data were randomly divided into two data sets, of which 70% were used to create the model and the remaining 30% were used to test the model. The relationship between skin temperature and multiple environmental parameters was determined by linear regression analysis in Matlab (2019a, MathWorks, Natick, MA, USA). The determination coefficient (R^2^) was used to evaluate the predictive performance of the model. The model was validated using the skin temperature data in the test set.

The statistical significance of the explanatory variable to the response variable was determined in the form of *p*-value. A small *p*-value indicated that the corresponding explanatory variable had a high statistical significance. When *p* < 0.001, the investigated parameter was considered to be highly significant.

#### 2.3.4. Stress Categories (Thresholds)

The threshold was the category of heat stress level that causes loss of animal production. The stress threshold categories are mild, moderate, severe and urgent. Mellado et al. [49] used THI [10] to analyze the pregnancy rate of sows. In their study, when THI < 74, the pregnancy rate was 93%; when 74 < THI < 78, the pregnancy rate was 91.8%; when 78 < THI < 82, the pregnancy rate was 91.4%; when THI > 82, the sow pregnancy rate was 89.8%. Following the categorization from the study of Mellado et al. [49], heat stress level can be classified as follows: THI < 74 indicates an appropriate environmental level, 74 ≤ THI < 78 indicates mild thermal stress, 78 ≤ THI < 82 indicates moderate thermal stress and THI ≥ 82 indicates severe thermal stress. A relationship between THI and ETIS has been established, and the ETIS defines heat stress thresholds according to the fitting equation.

#### 2.3.5. Comparative Analysis of Various Thermal Indices 

The ETIS thermal index model was compared with models such as THI [9,10,11,12,13,14,15,16], BGHI [17,18], ET [19] and H [33]. Pearson correlation coefficients between the thermal index and the selected physiological parameters were calculated. The larger the correlation coefficient, the better the prediction of the thermal index [21].

## 3. Results

### 3.1. Experimental Data Reliability Verification

#### 3.1.1. Experimental Data

The summarized dataset of air temperature, humidity, air velocity, skin temperature, respiration rate and core temperature are given in Table 1. 

#### 3.1.2. The Relationship between Temperature and Humidity Index and Core Temperature

**Figure 2:**
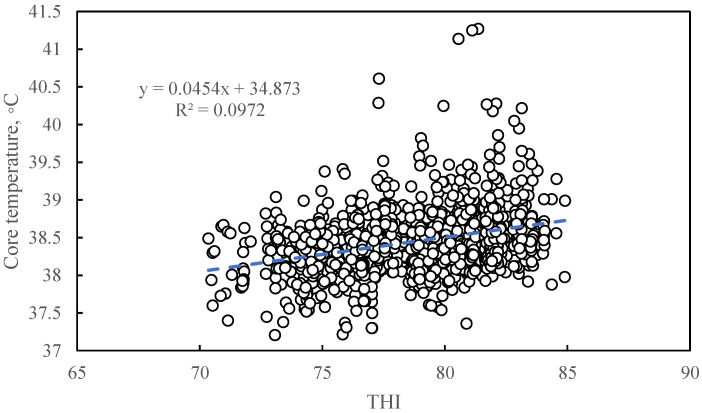
The correlations between temperature and humidity index and core temperature. The THI value was calculated based on the experimental data of 1029 sets of air temperature and relative humidity. The dots represent the scatter plot of the core temperature of the sow corresponding to the THI. The dotted line represents the model estimate based on the linear regression of sow core temperature with THI. The linear regression equation here is: y = 0.0454x + 34.873 (where y stands for core temperature (°C) and x stands for THI) (R^2^ = 0.0972, *p* < 0.0001).

Figure 2 shows the correlations between THI and core temperature. The relationship between THI and core temperature can be described by a linear regression equation: y = 0.0454x + 34.873 (where y stands for core temperature (°C) and x stands for THI) (R^2^ = 0.0972, *p* < 0.0001). 

#### 3.1.3. The Relationship between Temperature and Humidity Index and Respiration Rate

**Figure 3:**
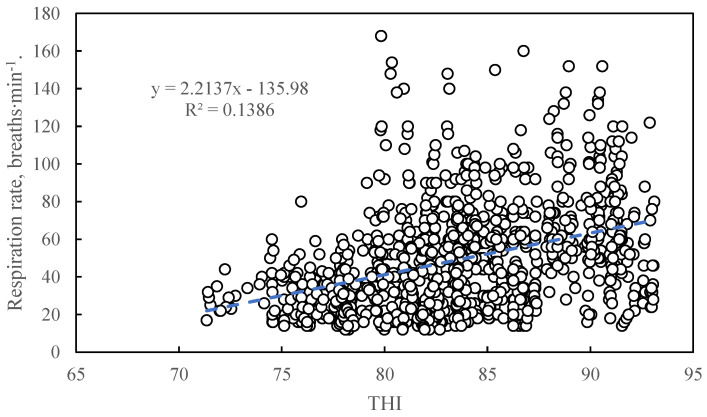
The correlation between temperature and humidity index and respiration rate. The THI value was calculated based on the experimental data from 1029 sets measuring air temperature and relative humidity. The dots represent the scatter plot of the respiration rate of the sow corresponding to the THI. The dotted line represents model estimate based on the linear regression of sow respiration rate with THI. The linear regression equation here is: y = 2.2137x − 135.98 (where y stands for respiration rate (breaths·min^−1^) and x stands for THI) (R^2^ = 0.1386, *p* < 0.0001).

Figure 3 shows the correlation between THI and respiration rate. The relationship between THI and respiration rate can be described by a linear regression equation: y = 2.2137x − 135.98 (where y stands for respiration rate (breaths·min^−1^) and x stands for THI) (R^2^ = 0.1386, *p* < 0.0001). 

#### 3.1.4. The Relationship between Temperature and Humidity Index and Skin Temperature

**Figure 4:**
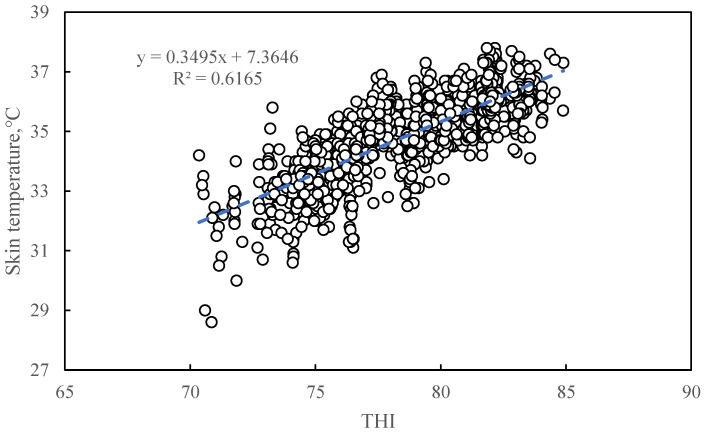
The correlation between temperature and humidity index and sow skin temperature. The THI value was calculated based on the experimental data from 1029 sets measuring air temperature and relative humidity. The dots represent the scatter plot of the skin temperature of the sow corresponding to the THI. The dotted line represents the model estimate based on the linear regression of sow skin temperature with THI. The linear regression equation here is: y = 0.3495x + 7.3646 (where y stands for skin temperature (°C) and x stands for THI) (R^2^ = 0.6165, *p* < 0.0001).

Figure 4 shows the correlation between THI and sow skin temperature. The relationship between THI and skin temperature can be described by a linear regression equation: y = 0.3495x +7.3646 (where y stands for skin temperature (°C) and x stands for THI) (R^2^ = 0.6165, *p* < 0.0001). 

### 3.2. Development of the Equivalent Temperature Index for Sows Model

**Figure 5:**
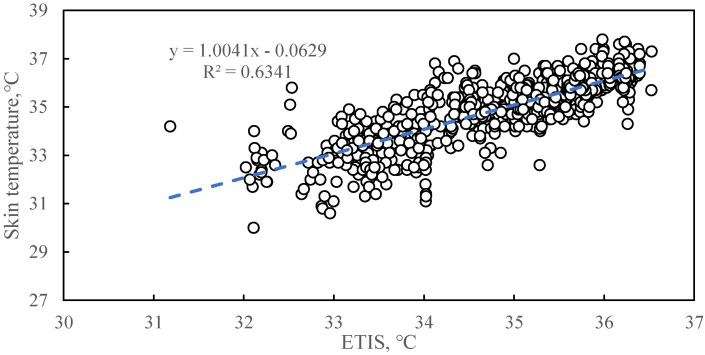
Regression of equivalent temperature index for sows to skin temperature based on training data. The dots represent the scatter plot of the skin temperature of the sow corresponding to the ETIS. The dotted line represents the model estimate based on the linear regression of sow skin temperature with 720 ETIS training data. The linear regression equation here is: y = 1.0041x − 0.0629 (where y stands for skin temperature (°C) and x stands for ETIS (°C)) (R^2^ = 0.6341, *p* < 0.0001).

Equation (10) was used to perform multiple linear regression in Matlab. Then, the estimated ratios of T, 50·T, RH·T, u^0.6827^·(T_S_ − T), 38, T, (38 + 273.15)^4^ and (T + 273.15)^4^ were obtained; these ratios were 0, 0.1152, 0.0006, −0.3132, 0, 0, 2.9370 × 10^−8^ and −4.8957 × 10^−8^, respectively. To be as consistent as possible with the definition of ETIS, the final ETIS can be determined as Equation (13). Figure 5 shows the correlation between ETIS and sow skin temperature.

(13) $$\begin{array}{l} \mathrm{ETIS}=T+0.0006\cdot \left(\mathrm{RH}-50\right)\cdot T-0.3132\cdot u^{0.6827}\cdot \left(38-T\right)-4.79\cdot \left(1.0086\cdot 38-T\right)+4.8957\cdot 10^{-8}\\ \;\;\;\;\;\;\cdot \left({\left(38+273.15\right)}^{4}-{\left(T+273.15\right)}^{4}\right) \end{array}$$

### 3.3. Validation of the Equivalent Temperature Index for Sows Model

**Figure 6:**
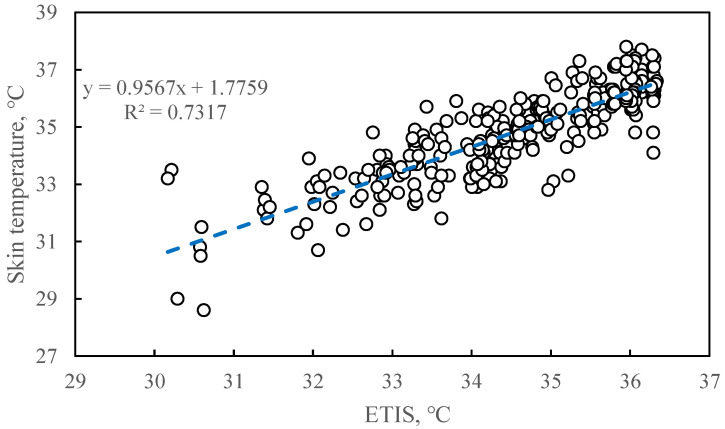
Regression of equivalent temperature index for sows to skin temperature based on test data. The dots represent the scatter plot of the skin temperature of the sow corresponding to the ETIS. The dotted line represents the model estimate based on the linear regression of sow skin temperature with 309 ETIS testing data. The linear regression equation here is: y = 0.9567x + 1.7759 (where y stands for skin temperature (°C) and x stands for ETIS (°C)) (R^2^ = 0.7317, *p* < 0.0001).

Figure 6 shows a scatter plot of the test data set of ETIS versus skin temperature. The measured coefficient of the skin temperature (R^2^) was 0.7317. This shows that the model derived from the training set can well estimate the skin temperature of the test data.

### 3.4. Classification of Heat Stress Threshold Based on Equivalent Temperature Index for Sows

**Figure 7:**
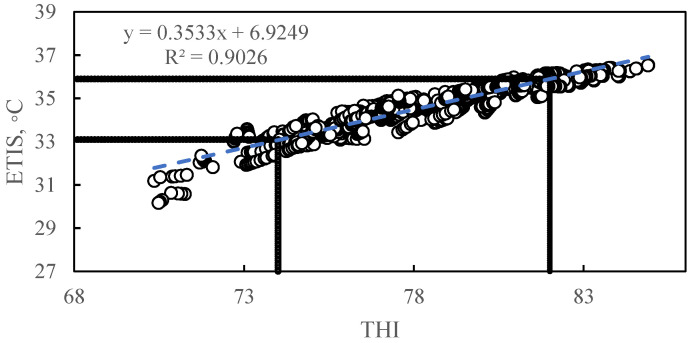
Regression on temperature and humidity index versus equivalent temperature index for sows. The dots represent the scatter plot of the ETIS corresponding to the THI. The dotted line represents model estimate based on linear regression of ETIS with THI. The linear regression equation here is: y = 0.3533x + 6.9249 (where y stands for ETIS (°C) and x stands for THI) (R^2^ = 0.9026, *p* < 0.0001).

Figure 7 shows a scatterplot of THI versus ETIS, and inductive regression equation is described: y = 0.3533x − 6.9249 (where y stands for ETIS (°C) and x stands for THI) (R^2^ = 0.9026, *p* < 0.0001).

Based on the linear regression model in Figure 7, heat stress threshold for ETIS can be developed based on thresholds developed for THI. The categories are as follows: ETIS < 33.1 °C is considered to be suitable, 33.1 °C ≤ ETIS < 34.5 °C is considered to be mild, 34.5 °C ≤ ETIS < 35.9 °C is considered to be moderate, and ETIS ≥ 35.9 °C is considered to be severe. As shown in Table 2, from the heat stress zone of ETIS, it can be concluded that the sow Farm of China Feed Research Center, located in North China, falls mostly in the mild and moderate heat stress range during the summer period. 

### 3.5. Comparison of Equivalent Temperature Index for Sows with Other Indices

ETIS was compared with other environmental thermal indices [9,10,11,12,13,14,15,16,17,18,19,21], and Pearson correlation coefficients were calculated to determine the relationship between selected physiological responses (skin temperature) and various thermal indices. As shown in Table 3, the thermal index positively correlated with the physiological response. 

## 4. Discussion

### 4.1. Experimental Data

It can be seen from the data that the highest temperature in the summer environment (34 °C) exceeded the upper limit of comfort for sows [7], but the heat dissipation process of the sow is not only related to the ambient temperature [22,23,29], and more factors should be considered overall.

### 4.2. The Relationship between THI and Physiological Parameters

#### 4.2.1. The Relationship between THI and Core Temperature

The coefficient of determination (R^2^) between THI and core temperature was 0.0972, which means that 9.7% of the core temperature change can be explained by the variation in THI. Sows are warm-blooded animals, and their rectal temperatures are relatively stable under normal conditions. Previous studies have shown that when the air temperature is above 25 °C, every 1 °C increase in air temperature will increase the rectal temperature by 0.099 °C [50]. However, conventional rectal thermometers are not precise enough to measure 0.099 °C, and minor changes cannot be accurately monitored [51]. Previous studies on the effect of air temperature on rectal temperature vary widely [50]. It is reasonable that the correlation between THI and core temperature is low.

#### 4.2.2. The Relationship between THI and Respiration Rate

The coefficient of determination (R^2^) between THI and respiration rate was 0.1386, which means that 13.9% of the respiration rate change can be explained by the variation in THI. As sows have fewer sweat glands, they dissipate differently from other animals. Meanwhile, sows are restricted by crates, and they recline or lie most of the time. When the sow reclines, it often alternates between being awake and asleep, which affects the sow’s breathing status [52]. The breathing status includes deep breathing and non-deep breathing. Under the same thermal conditions, if the sow breathes the same amount of air, the frequency between deep breathing and non-deep breathing will be different [53]. Individual differences and the lying position may also have an impact on a sow’s breathing. In previous studies, the monitored frequency of respiration rate was low [5], or the number of samples was small [51], so that the influences of the above factors were ignored. As an environmental thermal index, THI only includes air temperature and relative humidity. Relief of heat stress is essentially a process of heat dissipation, and air temperature and relative humidity are only part of the parameters of the heat dissipation process. As a result, it can be predicted that the correlation between THI and respiration rate is weak. The core temperature change is usually small, and it is also not suitable to be a crucial variable of the thermal index.

#### 4.2.3. The Relationship between THI and Skin Temperature

The coefficient of determination (R^2^) between THI and sow skin temperature was 0.6165, which means that 61.7% of the skin temperature change can be explained by the variation in THI. Since the sow’s skin is directly exposed to the air, skin temperature is greatly affected by the environment. Apart from the influence of external conditions, skin temperature is also affected by the sow’s internal heat production. The skin of the sow is the main channel for heat exchange. Skin temperature is the result of heat production and environmental factors [29]. Compared with other physiological parameters, skin temperature has the highest correlation with THI. The respiration rate changes after receiving the thermal sensory signal provided by the brain of the sow. The respiration rate is also affected by the state of sleep [52]. This process can inevitably have a delay compared to the skin. Since sows are warm-blooded animals, whose core temperature tends to be stable, the correlation between core temperature and thermal index is weak. Therefore, skin temperature is more suitable as the physiological response variable in the ETIS model.

### 4.3. Development and Verification of the ETIS Model

Each coefficient in ETIS represents the contribution or weight of each equivalent temperature to heat stress. The coefficient of determination (R^2^) between ETIS and sow skin temperature was 0.6341. The ETIS equation predicts the skin temperature of 63% of sows using the training data set. Skin temperature is not only affected by the environment but also by the sow’s internal factors, and there are individual differences among different sows. The ETIS model can predict 73% of skin temperature changes using the test data set. Therefore, the ETIS model construction can be considered reasonable.

### 4.4. Classification of Heat Stress Threshold Based on ETIS

In addition to the influence of air velocity, ETIS also includes the influence of air temperature and relative humidity. Therefore, the correlation between ETIS and THI is relatively high. According to the threshold partition of THI and the correlation equation between ETIS and THI, the threshold of ETIS can be determined. However, the balance between heat dissipation and heat production is not only affected by environmental parameters, but also by other factors related to the sow itself and management, such as sow genotype, hair thickness, health status, productivity level, activity level, etc. These animal or management-related factors change the range of threshold and heat stress categories.

### 4.5. Comparison of ETIS with Other Indices

The prediction of skin temperature was better with ETIS compared to other indices. In a hot environment, sows will not only be affected by air temperature and relative humidity, but also by air velocity. When the air temperature is lower than the skin temperature of the sow, increasing the air velocity is beneficial to alleviate heat stress. The main reason is that increasing the air velocity can increase the convective heat transfer coefficient of the sow, which is beneficial to increase the convective heat transfer of the sow. Compared with THI, BGHI and H, ETIS has an air velocity term. ET simply combines air temperature, relative humidity and air velocity, and it uses a small amount of test data based on sensible heat dissipation of 3.4–70 kg pig [19]. Sensible heat is an indirect measurement, so errors will inevitably occur. Moreover, large pigs tend to avoid heat, and little pigs tend to avoid cold. The characteristic lengths of big pigs and small pigs are different, so the heat dissipation characteristics will be slightly different. Therefore, ETIS is significantly better than ET for predicting the skin temperature of sows.

The skin is a window for heat exchange between the sow and the external environment. When the thermal environment is severe, the sow skin is stimulated at first. ETIS can reasonably predict the influence of the environment on the sow skin temperature, so it can be considered that the ETIS model can be used to evaluate heat stress level of sows. However, it should be noted that the index is created in a fully enclosed sow barn, so the applicable conditions should be similar. Sows have individual differences, and sows in different regions have different environmental adaptations. These will affect the use of ETIS.

### 4.6. Summary of the Study and Research Perspectives

In this study, by considering the influencing factors of air temperature, relative humidity and air velocity, according to the law of heat transfer between the sow and the surrounding environment, the sows equivalent temperature model was integrated. The equivalent temperature and the sow skin temperature were used in the multiple linear regression analysis to determine the unknown coefficients of the sow equivalent model species, and to finally determine the ETIS equation. The ETIS equation has a good correlation with THI. THI is used to partition the THI thermal threshold. The classification is as follows: suitable temperature ETIS < 33.1 °C, mild temperature 33.1 °C ≤ ETIS < 34.5 °C, moderate stress temperature 34.5 °C ≤ ETIS < 35.9 °C, and severe temperature ETIS ≥ 35.9 °C. ETIS was also compared with other various indices. Finally, ETIS was concluded to have the highest correlation with skin temperature.

In this study, the correlation between ETIS and THI was used to divide the ETIS index. However, under actual conditions, the living environment of sows is different, and the characteristics of heat discomfort should be inconsistent. The thermal threshold in different regions should be slightly different. Thus, we must proceed with future studies. The actual production data will be used to verify the ETIS thermal stress threshold or make some corrections to the ETIS thermal stress threshold.

## 5. Conclusions

The following conclusions can be drawn from this study:

(1) A thermal index model called ETIS was developed. This model was used to predict the level of heat stress in sows. The thermal index takes into account the heat transfer characteristics of the sows. The correlation between the ETIS index and THI index (R^2^) was 0.90, and the correlation with sow skin temperature (R^2^) was 0.67.

(2) The ETIS heat stress threshold was classified according to the threshold defined by THI. The classification was as follows: suitable temperature ETIS < 33.1 °C, mild temperature 33.1 °C ≤ ETIS < 34.5 °C, moderate stress temperature 34.5 °C ≤ ETIS < 35.9 °C, and severe temperature ETIS ≥ 35.9 °C.

(3) Compared with other thermal indexes, the ETIS model has the best prediction of skin temperature (R = 0.82).

## Figures and Tables

**Table 1:** Statistics of the integrated dataset. N represents the total amount of data. SD represents the standard deviation of the data.

Item	N	Mean	SD	Maximum	Minimum
Air temperature (T), °C	1029	28.7	2.6	34.0	21.9
Relative humidity (RH), %	1029	65.8	10.0	89.8	40.4
Air velocity (u), m·s^−1^	1029	0.07	0.07	0.29	0.00
Skin temperature ($T_{s}$), °C	1029	34.9	1.4	37.8	28.6
Respiration rate (RR), breaths·min^−1^	1029	49	28	168	12
Core temperature (T_c_), °C	1029	38.44	0.46	41.27	37.21

**Table 2:** ETIS heat stress threshold. Threshold distribution of ETIS was determined according to the threshold distribution of THI and the correlation equation between THI and ETIS.

Category	THI	ETIS, °C
Suitable	THI < 74	ETIS < 33.1
Mild	74 ≤ THI < 78	33.1 ≤ ETIS < 34.5
Moderate	78 ≤ THI < 82	34.5≤ ETIS < 35.9
Severe	82 ≤ THI	35.9 ≤ ETIS

Note: THI is temperature and humidity index. ETIS is the equivalent temperature index for sows.

**Table 3:** Pearson correlation coefficient between the thermal index and physiological responses (skin temperature) (all *p*-values < 0.0001). The thermal index and the skin temperature are positively correlated. When the Pearson correlation coefficient R is closer to 1, the correlation is stronger.

Thermal Index	Equation	Skin Temperature Correlation (R)
ETIS	$\begin{array}{l} \mathrm{ETIS}=T+0.0006\cdot \left(\mathrm{RH}-50\right)\cdot T-0.3132\cdot u^{0.6827}\cdot \left(38-T\right)-4.79\\ \;\;\;\;\;\;\cdot \left(1.0086\cdot 38-T\right)+4.8957\cdot 10^{-8}\cdot \left({\left(38+273.15\right)}^{4}-{\left(T+273.15\right)}^{4}\right) \end{array}$	0.82
THI [9]	$\mathrm{THI}=T+0.36\cdot T_{\mathrm{wb}}+41.5$	0.78
THI [10]	$\mathrm{THI}=0.8\cdot T+\left(\frac{\mathrm{RH}\cdot \left(T-14.4\right)}{100}\right)+46.4$	0.79
THI [11]	$\mathrm{THI}=0.65\cdot T+0.35\cdot T_{\mathrm{wb}}$	0.79
THI [12]	$\mathrm{THI}=T^{{}^{\circ}}-\left(0.55-0.0055\cdot \mathrm{RH}\right)\cdot \left(T^{{}^{\circ}}-58\right)$	0.79
THI [13]	$\mathrm{THI}=0.72\cdot T+0.72\cdot T_{\mathrm{wb}}+40.6$	0.78
THI [16]	$\mathrm{THI}=\left(1.8\cdot T+32\right)-\left[0.55\cdot \left(\frac{\mathrm{RH}}{100}\right)\right]\cdot \left[\left(1.8\cdot T+32\right)-58\right]$	0.69
THI [14]	THI=T−(0.55−0.0055·RH)·(T−14.5)	0.79
THI [15]	$\mathrm{THI}=0.27\cdot T+1.35\cdot T_{\mathrm{wb}}+34.07$	0.73
BGHI [17]	$\mathrm{BGHI}=T_{g}+0.36\cdot T_{\mathrm{dp}}+41.5$	0.71
ET [19]	$\mathrm{ET}=T+0.0015\cdot \left(\mathrm{RH}-50\right)\cdot T+\left[-1.0\cdot 42-T\cdot \left(v^{0.66}-0.2^{0.66}\right)\right]$	0.75
H [21]	H=1.006·T+RHPm·10(7.5·T273.3+T)·(71.28+0.052·T)	0.68

Note: ETIS is the equivalent temperature index for sows. THI is temperature and humidity index. BGHI is the globe-humidity index. ET is the effective temperature. H is the enthalpy (kJ·kg^−1^). T is the dry bulb temperature (℃). T° is the dry bulb temperature(°F). T_g_ is the black globe temperature (℃). T_wb_ is the wet bulb temperature (℃); T_dp_ is the dew point temperature (℃). RH is the relative humidity (%). P_m_ is high mercury of barometric pressure (mmHg).

## Data Availability

All data sets during the current study are available from the corresponding author on fair request.

## References

[1] Barb C.R., Estienne M.J., Kraeling R.R., Marple D.N., Rampacek G.B., Rahe C.H., Sartin J.L. (1991). Endocrine changes in sows exposed to elevated ambient temperature during lactation. Domest. Anim. Endocrinol..

[2] Almond P.K., Bilkei G. (2005). Seasonal infertility in large pig production units in an Eastern-European climate. Aust. Vet. J..

[3] Bloemhof S., Mathur P.K., Knol E.F., van der Waaij E.H. (2013). Effect of daily environmental temperature on farrowing rate and total born in dam line sows. J. Anim. Sci..

[4] Tummaruk P., Tantasuparuk W., Techakumphu M., Kunavongkrit A. (2004). Effect of Season and Outdoor Climate on Litter Size at Birth in Purebred Landrace and Yorkshire Sows in Thailand. J. Vet. Med Sci..

[5] Quiniou N., Noblet J. (1999). Influence of high ambient temperatures on performance of multiparous lactating sows. J. Anim. Sci..

[6] Black J.L., Mullan B.P., Lorschy M.L., Giles L.R. (1993). Lactation in the sow during heat stress. Livest. Prod. Sci..

[7] D’Allaire S., Drolet R., Brodeur D. (1996). Sow mortality associated with high ambient temperatures. Can. Vet. J..

[8] St-pierre N.R., Cobanov B., Schnitkey G. (2003). Economic Losses from Heat Stress by US Livestock Industries1. J. Dairy Sci..

[9] Thom E.C. (1958). Cooling degrees-days air conditioning, heating, and ventilating. Trans. ASAE.

[10] Thom E.C. (1959). The discomfort index. Weatherwise.

[11] Ingram D.L. (1965). The effect of humidity on temperature regulation and cutaneous water loss in the young pig. Res. Vet. Sci..

[12] Bond C.F.K.T.E. (1971). Bioclimatic Factors and Their Measurements. A Guide to Environmental Research in Animals.

[13] Maust L.E., Mcdowell R.E., Hooven N.W. (1972). Effect of Summer Weather on Performance of Holstein Cows in Three Stages of Lactation. J. Dairy Sci..

[14] NOAA (National Oceanic and Atmospheric Administration) (1976). Livestock Hot Weather Stress.

[15] Fehr R.L., Priddy K.T., Mcneill S.G., Overhults D.G. (1983). Limiting Swine Stress with Evaporative Cooling in the Southwest. Trans. ASAE.

[16] Wu Z., Chen Z., Zang J., Wang M., Yang H., Ren F., Liu J., Feng G. (2018). Cooling performance of wet curtain fan-fabric duct ventilation system in house of pregnant sows. Trans. Chin. Soc. Agric. Eng..

[17] Buffington D.E., Collazo-Arocho A., Canton G.H., Pitt D., Thatcher W.W., Collier R.J. (1981). Black globe-humidity index (bghi) as comfort equation for dairy cows. Trans. ASAE.

[18] Júnior G.M.d.O., Ferreira A.S., Oliveira R.F.M., Silva B.A.N., de Figueiredo E.M., Santos M. (2011). Behaviour and performance of lactating sows housed in different types of farrowing rooms during summer. Livestock Sci..

[19] Bjerg B., Rong L., Zhang G. (2018). Computational prediction of the effective temperature in the lying area of pig pens. Comput. Electron. Agric..

[20] Bjerg B.S., Kai P. CFD Prediction of Heat Transfer in Heated or Cooled Concrete Floors in Laying Areas for Pig. Proceedings of the 2019 ASABE Annual International Meeting.

[21] Rodrigues V.C., da Silva I.J.O., Vieira F.M.C., Nascimento S.T. (2011). A correct enthalpy relationship as thermal comfort index for livestock. Int. J. Biometeorol..

[22] Yunus A.C.D., Afshin J.G. (2014). Heat and Mass Transfer: Fundamentals and Applications.

[23] Modest M.F. (2013). Radiative Heat Transfer.

[24] Marple D.N., Jones D.J., Alliston C.W., Forrest J.C. (1974). Physiological and endocrinological changes in response to terminal heat stress in swine. J. Anim. Sci..

[25] Patience J., Chaplin R. (1991). Physiological and nutritional effects of heat-stress in the pig. FASEB J..

[26] Patience J.F., Umboh J.F., Chaplin R.K., Nyachoti C.M. (2005). Nutritional and physiological responses of growing pigs exposed to a diurnal pattern of heat stress. Livest. Prod. Sci..

[27] Ross J.W., Hale B.J., Gabler N.K., Rhoads R.P., Keating A.F., Baumgard L.H. (2015). Physiological consequences of heat stress in pigs. Anim. Prod. Sci..

[28] Ross J.W., Hale B.J., Seibert J.T., Romoser M.R., Adur M.K., Keating A.F., Baumgard L.H. (2017). Physiological mechanisms through which heat stress compromises reproduction in pigs. Mol. Reprod. Dev..

[29] Bond T.E., Kelly C.F., Heitman A.H. (1959). Hog house air conditioning and ventilation data. Trans. ASAE.

[30] Bianca W. (1976). The signifiance of meterology in animal production. Int. J. Biometeorol..

[31] Parsons K. (2014). Human Thermal Environments: The Effects of Hot, Moderate, and Cold Environments on Human Health, Comfort, and Performance.

[32] Beckett F.E. (1965). Effective temperature for evaluating or designing hog environments. Trans. ASAE.

[33] Bergman T.L., Lavine A.S., Incropera F.P., DeWitt D.P. (2011). Fundamentals of Heat and Mass Transfer.

[34] Kreith F., Bohn M. (2010). Principles of Heat Transfer.

[35] Wang X.S., Gao H.D., Gebremedhin K.G., Bjerg B.S., Van Os J., Tucker C.B., Zhang G.Q. (2018). A predictive model of equivalent temperature index for dairy cattle (ETIC). J. Therm. Biol..

[36] Li H., Rong L., Zhang G. (2016). Study on convective heat transfer from pig models by CFD in a virtual wind tunnel. Comput. Electron. Agric..

[37] Wang X.S., Zhang G.Q., Choi C.Y. (2018). Effect of airflow speed and direction on convective heat transfer of standing and reclining cows. Biosyst. Eng..

[38] Gravas L. (1981). The exercise needs for tied and free-moving dry sows. Appl. Anim. Ethol..

[39] Massabie P., Granier R. Effect of Air Movement and Ambient Temperature on the Zootechnical Performance and Behavior of Growing-Finishing Pigs. Proceedings of the the 94th ASAE Annual International Meeting.

[40] Cao M.B., Zong C., Wang X.S., Teng G.H., Zhuang Y.R., Lei K.D. (2020). Numerical simulations of airflow and convective heat transfer of a sow. Biosyst. Eng..

[41] Bruce J.M., Clark J.J. (1979). Models of heat production and critical temperature for growing pigs. Anim. Prod. Sci..

[42] Collier R.J., Gebremedhin K.G. (2014). Thermal Biology of Domestic Animals. Annu. Rev. Anim. Biosci..

[43] Hwang R.L., Lin T.P., Matzarakis A. (2011). Seasonal effects of urban street shading on long-term outdoor thermal comfort. Build. Environ..

[44] Kantor N., Unger J. (2011). The most problematic variable in the course of human-biometeorological comfort assessment—The mean radiant temperature. Cent. Eur. J. Geosci..

[45] Kaynakli O., Kilic M. (2005). Investigation of indoor thermal comfort under transient conditions. Build. Environ..

[46] Langner M., Scherber K., Endlicher W.R. (2013). Indoor heat stress: An assessment of human bioclimate using the UTCI in different buildings in Berlin. ERDE.

[47] Matzarakis A., Amelung B. (2008). Physiological Equivalent Temperature as Indicator for Impacts of Climate Change on Thermal Comfort of Humans. Seasonal Forecasts, Climatic Change and Human Health.

[48] Olesen B.W., Parsons K.C. (2002). Introduction to thermal comfort standards and to the proposed new version of EN ISO 7730. Energy Build..

[49] Mellado M., Gaytán L., Macías-Cruz U., Avendaño L., Meza-Herrera C., Lozano E.A., Rodríguez Á., Mellado J. (2018). Effect of climate and insemination technique on reproductive performance of gilts and sows in a subtropical zone of Mexico. Austral. J. Vet. Sci..

[50] Bjerg B., Brandt P., Pedersen P., Zhang G. (2020). Sows’ responses to increased heat load—A review. J. Therm. Biol..

[51] Cabezón F., Schinckel A.P., Marchant-Forde J.N., Johnson J.S., Stwalley R.M. (2017). Effect of floor cooling on late lactation sows under acute heat stress. Livestock Sci..

[52] Li W., Yang X.D., Dai A.N., Chen K. (2018). Sleep and Wake Classification Based on Heart Rate and Respiration Rate. IOP Conf. Ser. Mater. Sci. Eng..

[53] Cheng K.S., Lee P.F. (2018). A Physiological/Model Study on the Effects of Deep Breathing on the Respiration Rate, Oxygen Saturation, and Cerebral Oxygen Delivery in Humans. Neurophysiology.

